# Effectiveness of Targeted Advisory Interventions in Enhancing Welfare on Dairy Farms

**DOI:** 10.3390/ani15152197

**Published:** 2025-07-25

**Authors:** Susy Creatini, Cristina Roncoroni, Federica Salari, Iolanda Altomonte, Giovanni Brajon, Mina Martini

**Affiliations:** 1 Department of Veterinary Science, Università di Pisa, Viale delle Piagge 2, 56124 Pisa, Italy; susy.creatini@phd.unipi.it (S.C.); mina.martini@unipi.it (M.M.); 2 Istituto Zooprofilattico Sperimentale del Lazio e Della Toscana “M. Aleandri”, Via Appia Nuova 1411, 00178 Rome, Italy; cristina.roncoroni@izslt.it; 3 Istituto Zooprofilattico Sperimentale del Lazio e Della Toscana “M. Aleandri”, Via Castelpulci 43, 50018 Florence, Italy; giovanni.brajon@izslt.it

**Keywords:** cow welfare, dairy farm, farm management, targeted consultancy

## Abstract

Animal welfare assessments have raised farmers’ awareness of their management practices, contributing to measurable improvements. However, these protocols often highlight critical points without providing clear guidance on the prioritization of corrective actions. To address this gap, qualified advisory support may play a pivotal role in translating assessments into effective and sustainable interventions. This study evaluates the impact of direct and continuous expert support on improving animal welfare in dairy farms. Data were collected from 21 dairy farms in southeastern Tuscany (Italy) using the Classyfarm (CReNBA) protocol. Each farm underwent two assessments at a three-month interval (T0 and T1), during which tailored support was provided to address specific criticalities. At T0, over 60% of the farms obtained only marginally acceptable welfare scores (mean 67.48 ± 4.75), with major deficiencies in farm management practices, particularly regarding hygiene and space management. At T1, all farms showed substantial improvements, with an average increase of 22% in total welfare scores (mean 82.05 ± 5.71) and a mean of nine improved parameters per farm. These findings underscore the effectiveness of structured, continuous consultancy in not only resolving critical issues but also in fostering more informed, proactive, and sustainable farm management. The direct involvement of experts appears to be a key driver in enhancing both animal welfare and operational outcomes in dairy farming.

## Simple Summary

This study assessed animal welfare on 21 dairy farms in Tuscany, Italy, to examine the impact of expert support on farm conditions. The initial evaluation (T0) revealed several welfare-related issues, such as management practices, facilities, and animal-based measures, with many farms failing to reach sufficient welfare levels. After three months of targeted support, significant improvements were observed. In the second evaluation (T1), all farms showed improvements in management, hygiene, resting space, hoof disease prevention, and access to clean water. The study highlights the importance of ongoing collaboration between farmers and experts to ensure long-term sustainability and the enhancement of animal welfare.

## 1. Introduction

The introduction of standardized protocols for animal welfare assessment has contributed to increasing farmers’ awareness of their farming conditions, promoting significant improvements over time [1]. However, despite the valuable information and feedback these protocols provide, farms often require additional support to identify specific actions to meet welfare criteria effectively [1].

Numerous studies indicate that animal welfare is a priority for dairy farmers. However, several obstacles hinder its effective implementation, including economic difficulties, time constraints [2,3,4], labor shortages, and regulatory pressures [5,6,7]. These factors not only limit the adoption of improvement measures but can also increase stress among farm operators, negatively impacting animal welfare [8]. In this context, professional support and collaborative networks between farmers and experts emerge as key strategies to improve farm conditions [9]. Indeed, a high level of external support is associated with better animal welfare standards [10,11], enhanced farm management efficiency and sustainability [12], and greater work manageability for farmers [13].

Croyle et al. [1] emphasized that farmers prefer to address animal welfare challenges through a collaborative approach with other producers and specialized consultants. This shared model fosters collective responsibility in decision-making and goal setting [14], transforming farmers into aware and active participants in defining improvement strategies [15]. Such an approach is crucial to counteract *barn blindness* or *basal shift syndrome* (BSS), where farmers become desensitized to suboptimal housing conditions and start perceiving problematic situations as normal [1,16]. Moreover, while assessment protocols are useful for risk identification, they often isolate issues without considering the complexity of the entire management system, making it difficult to prioritize interventions. To address these limitations, a more integrated and structured approach is needed—one that combines welfare assessment with continuous and targeted advisory support to guide farmers in decision-making and implementation. This study investigates the added value of continuous expert support in improving animal welfare on dairy farms. Conducted within a dairy producer consortium, it involves a sample of farms homogeneous in production type but varying in baseline welfare levels. Expert consultancy was provided through periodic visits, training activities, and personalized assistance aimed at defining specific, measurable interventions. By comparing initial assessments (T0) with those after targeted support (T1), the study evaluates whether ongoing consultancy can facilitate problem identification, stimulate managerial changes, and enhance overall animal welfare.

Unlike previous studies focusing solely on evaluation protocols, this research highlights the fundamental role of continuous technical support as a key driver of change. Similar evidence from other contexts supports this: for example, Danish studies have demonstrated that collaborative and transdisciplinary advisory approaches improve knowledge sharing and the effectiveness of welfare strategies [17]. Research on veterinary consultancy also shows that farmers prefer ongoing, practical support rather than isolated problem diagnoses, which enhances their satisfaction and readiness to adopt recommendations [18]. These findings reinforce the importance of continuous expert support for sustainable welfare improvements and provide valuable insights for supply chain policies aiming to integrate structured consultancy into continuous improvement processes.

## 2. Materials and Methods

### 2.1. Study Design and Data Collection

Data collection was conducted between February and May 2023 on 21 dairy farms located in a high-milk-production area of Tuscany, Italy (approximately 42.85° N, 11.17° E). All farms were members of the same milk supply consortium and were selected on a voluntary basis. Importantly, the advisory service evaluated in this study was offered by the consortium to all its member farms as part of a broader welfare improvement initiative. Participation was not researcher-driven but rather left to the discretion of individual farmers. Of all eligible farms, 21 chose to participate, corresponding to 63.6%. Supplementary Table S1 provides the average characteristics of the farms under study, including total number of animals by category, average milk yield, type of feed, and housing system. Each farm underwent two animal welfare assessments (T0 and T1) at a three-month interval. During both surveys, the evaluation was conducted using the Classyfarm protocol for welfare assessment on a voluntary basis [19], that uses animal-based, management-based, and resource-based indicators to classify risks on livestock farms, developed by the National Reference Centre for Animal Welfare, funded by the Italian Ministry of Health. Using specific checklists, the Classyfarm system is designed to collect data, support official controls, promote the implementation of welfare levels, and provide information to consumers. Classyfarm is designed to identify negative aspects or deficiencies and classify farms based on welfare risk levels. The checklist used in this study was the “Check-list for Dairy Cows in Free-Stall Housing” [20], consisting of 105 evaluation items, divided into four areas: Area A—“Management and Personnel” (45 evaluation items); Area B—“Facilities and Equipment” (32 evaluation items); Area C—“Animal-Based Measures (ABMs)” (19 evaluation items); Area D—High-Risk Area (9 evaluation items). Items in the Classyfarm system’s checklists include two or three answer options: insufficient, acceptable, or excellent; and insufficient or acceptable [21,22]. All evaluations and data recordings were performed by a trained veterinarian, assisted by an experienced farm technician. Inter-rater reliability was ensured through training sessions, including concordance testing, to align assessors and standardize evaluations.

The initial screening (T0) enabled the early identification of major farm-specific critical issues, allowing for targeted interventions and raising farmers’ awareness of best management practices. The continuous and targeted support provided to farmers included regular on-farm visits, personalized consultations to address identified problems, assistance in planning and implementing corrective actions, and training sessions for farm personnel. Additionally, a monitoring system based on standardized checklists and periodic feedback was implemented to assess progress and ensure effective application of improved management practices.

Through a comprehensive analysis of farm-specific challenges, tailored intervention strategies were developed aimed at improving not only animal welfare but also management efficiency and resource optimization. The consultancy process was conducted using an integrated approach, which included direct observation and systematic recording of critical issues, as well as a detailed analysis of potential short-term modifications. Considering the three-month interval between assessments, interventions primarily focused on managerial and organizational aspects that could be feasibly implemented even in well-established farms. Continuous support included periodic on-site visits and telephone communications to monitor the implementation of interventions and assist in optimizing operational practices. Particular attention was given to selecting effective communication strategies tailored to the farmers’ profiles, in accordance with the methodological principles outlined by Croyle et al. [1]. To maximize the effectiveness of the advisory support, the consultancy protocol adopted prioritized interventions based on three key criteria: importance, urgency, and feasibility of corrective actions (Supplementary Table S2).

### 2.2. Data Processing and Statistical Analysis

Data from the Classyfarm report, expressed as total and partial scores for each area (in percentage), and insufficient evaluations, were analyzed to identify the main critical issues observed at T0 and the improvements recorded at T1, highlighting the most relevant problems common to all examined farms.

Particular attention was given to aspects with a significant impact on animal welfare, as defined during the development phase of the animal welfare protocol [23], to insufficient evaluations on criteria for which more than 20% of farms did not meet the minimum required scores, and to instances related to regulatory standards.

In this study, the acceptance threshold was set at a score of 60%, expressed as a percentage, which is considered the minimum acceptable level to ensure animal welfare.

To evaluate changes over time (T0 vs. T1), the normality of the data was previously assessed using the Shapiro–Wilk test. Since the data were not normally distributed, the Wilcoxon test was applied to compare the data over time (T0 vs. T1), with the significance level set at *p* ≤ 0.05.

## 3. Results

### 3.1. Overall Evaluation of the Farms Under Study

Total and partial mean scores for each area obtained during the first screening (T0) and the second screening (T1) are summarized in Table 1. At the first screening (T0), 71% of the farms scored between 60% and 70%, while 29% scored between 70% and 80%. Notably, 90% of the farms exhibited at least one insufficient evaluation in relation to legislative requirements [24,25].

At the second screening (T1), all farms showed improvements, with total scores increasing by over 20% and no deficiencies related to legislative compliance. These results confirm the effectiveness of the targeted advisory intervention. Only Area D—Major Risks did not show a statistically significant improvement (*p* = 0.4173), despite an average increase of 4%. Referring to the presence of emergency plans and alert systems, this area is crucial in case of significant exceptional events, but it is not included in the algorithm that returns the final total welfare score, as the measures it considers do not directly affect the daily welfare of the animals. Although the number of farms with scores below the minimum threshold (60%) decreased across all areas (Table 2), Area D addresses issues that require time and resources to resolve, limiting short-term progress.

The total scores achieved at T1 were satisfactory and aligned with national averages for the period: Area A—Management and Personnel: 69%; Area B—Structures and Facilities: 76%; Area C—Animal-Based Measures: 84%; Area D—Major Risks: 58% (reference date: 11 May 2023).

Although all the farms observed in the first screening (T0) scored a total of ≥60%, significant deficiencies were found in the individual areas. Table 2 reports the percentage of farms that did not achieve a sufficient score in each area. In fact, a significant reduction in the number of insufficient farms at T1 was observed across all areas. Notably, management and personnel (Area A) showed a marked improvement, with 43% of farms initially under 60% at T0, decreasing to 0% at T1. As for Areas B, C, and D at T0, with 33%, 10%, and 5% of farms failing to meet the required standards respectively, these percentages fell to 0% at T1.

### 3.2. Areas Results

The report analysis highlighted the most critical issues across the 21 farms included in the study. For each of the four assessed areas (Areas A, B, C, and D), the identified issues were reported, categorized by animal group and age class: animals over 6 months old (Table 3) and animals under 6 months old (Table 4).

Critical areas were defined as those where more than 20% of farms failed to meet the minimum requirements for specific indicators, legislative compliance, and/or criteria with a significant impact on animal welfare [23]. These areas received the greatest attention from consultants, who supported farm operators in addressing the identified issues.

## 4. Discussion

One of the most significant aspects emerging from the comparison between the first (T0) and the second observation (T1) was the substantial improvement recorded in farm management (Area A, +34%) and facilities (Area B, +26%). These results were directly influenced by consultancy support, which provided farmers with concrete tools to optimize daily animal management and enhance farm efficiency. The intervention of consultants had a decisive impact, facilitating the resolution of many initial issues and leading to tangible improvements. In particular, the collaboration between experts and farmers enabled better management of animal groups and more effective use of available space, all of which had a direct impact on animal welfare. A particularly relevant finding is that, despite farm structures already being operational and well-established, consultancy support enabled significant improvements without requiring, where possible, major structural modifications. This demonstrates that the optimization of management practices and increased farmer awareness can lead to substantial progress in animal welfare.

### 4.1. Improvements in Management

The most common issues identified at time T0 were primarily related to space management, hygiene conditions, and the quality of resting surfaces, all of which have a direct impact on animal comfort, productivity, and welfare. Optimizing bedding and resting surface management played a key role in increasing the Area A scores. It has been shown that dairy cows spend more time lying down when provided with thick [26], soft [27], and dry [28] bedding, with positive effects on preventing limb injuries [29] and improving milk production [30].

Hygiene management also proved to be a key factor: poor on-farm hygiene is strongly associated with the incidence of conditions such as mastitis [31] and hoof disorders [32], with consequences in terms of increased veterinary costs and reduced productivity [33]. Moreover, inadequate availability of proper resting areas compels animals to lie down in unsuitable spaces, further compromising body cleanliness [34]. In this study, the synergistic effect of these factors had a substantial impact on hygiene indicators: at T0, 100% of the farms showed inadequate animal cleanliness (Area C), indicating the need for targeted interventions. Consistently, over 60% of farms showed deficiencies in the availability of appropriate resting areas, and 24% did not meet hygiene standards for bedding and resting area management. Corrective actions recommended by consultants included more frequent replenishment of bedding with clean, dry straw, improved drainage management in resting areas, and the installation of elevated platforms or rubber mats in humid zones.

### 4.2. Improvements in Calf Welfare and Disease Prevention

The critical issues identified were not limited to adult animals but also affected calves under six months of age. At T0, 9.5% of farms showed deficiencies related to group housing space and bedding quality. According to EFSA [35], an adequate resting surface is essential to prevent slipping, falling, and stress, which can compromise the development and health of young animals. Additionally, substrate choice influences calf cleanliness, thermal comfort, and overall physiology [36], and providing clean, dry hay is essential to reduce the risk of gastrointestinal diseases [37]. Suggested corrective measures included regular supply of dry bedding, drainage improvement in pens, and the use of raised surfaces or perforated rubber mats to reduce moisture and enhance hygiene.

Another critical issue identified in young stock housing was the limited ability of calves to see and have physical contact with each other, which can cause isolation stress and lead to poor social behaviors [35]. Studies indicate that calves housed in pairs are less fearful, while isolated calves tend to exhibit higher levels of fear. Those housed in individual pens with physical contact show an intermediate response [38]. Consultants recommended the use of double pens or the modification of individual pens to allow at least visual and partial physical contact between calves.

### 4.3. Foot Disease Prevention

Another major concern was inadequate hoof disease prevention, observed in 61% of farms at T0. Limb disorders are a leading cause of pain and discomfort in dairy cattle. They negatively affect mobility, overall well-being, and productivity. Such disorders reduce the animal’s ability to walk, limiting proper feeding and social interactions. This decline impacts both quality of life and productive performance. Additionally, hoof problems increase the risk of infections and other complications, often requiring costly and long-term treatments [39,40,41]. Targeted consultancy support helped farmers develop more effective prevention plans, such as regular foot disinfection, the introduction of footbaths, the scheduling of hoof trimming plans, and improved flooring maintenance in the barns. These practices are crucial for reducing the incidence of hoof diseases, improving the overall health of the animals, and preventing serious issues like pododermatitis and laminitis. Additionally, the introduction of anti-slip flooring and the use of soft, dry bedding can help reduce the risk of limb injuries, thus enhancing the comfort and mobility of the animals.

Previous studies indicate that farmers often underestimate the severity of hoof disorders, focusing more on the visible and immediate problems of the animals rather than on prevention [42,43]. However, consultancy support helped bridge this gap, promoting a more proactive approach to hoof health management. Increased awareness among farmers regarding the importance of proper preventive management led to a reduction in the need for antibiotic treatments and an overall improvement in the herd’s health. The involvement of experts also facilitated farmer training on early monitoring techniques for hoof disorders, allowing them to intervene promptly and reduce the risk of serious complications.

### 4.4. Water Management

Cleaning of water points, which was insufficient in 38% of farms during the first screening, directly impacts water quality and palatability, affecting animal welfare, growth, and production [44]. Factors that can decrease water quality and palatability, and consequently reduce consumption, include microbial contamination, especially from fecal material [45,46], and temperature [47]. As highlighted by Schütz et al. [44], cows show a marked preference for clean drinking water, and a restriction in drinking behavior can have negative effects on production and animal welfare, also reducing feed intake [48,49], milk production, and body weight [49,50]. Recommended interventions included daily cleaning of water troughs, installation of drainage valves, and increasing the number of drinkers in high-density animal areas. In farms with low water pressure, pressure regulators and flow meters were advised. Restrictions in drinking behavior can also cause changes in behavior, such as increased aggression and the time spent around the drinking trough [50]. This element, along with the number of water troughs on the farm (insufficient in 38% of farms at T0) and overall water availability, which also takes into account the type of trough and the availability for all animals (insufficient in 10% of farms at T0, and acceptable for all others), plays a key role in the assessment, as it can influence one of the five freedoms underlying animal welfare: “freedom from thirst” [51].

### 4.5. Importance of a Dedicated Infirmary

Finally, the lack of an infirmary for sick or injured animals was identified as a shortcoming in 43% of farms. Providing a dedicated space for animal care is essential for effective disease management and infection control [52]. Consultants recommended and helped operators to identify a separate, well-ventilated, and easy-to-clean area, equipped with soft bedding and tools for individual health monitoring. These interventions improved animal health management and farm biosecurity.

### 4.6. Limitations and Applicability

This study provides insights into the improvement of animal welfare and farm management. Although the results cannot be considered fully representative of all dairy farms, the standardization of the sector allows for the application of the advisory approaches used in this study to other similar contexts. However, given that the sample of farms is relatively small and geographically limited, further research with larger samples and longer follow-up periods is needed to validate these results and assess their effectiveness in different settings. Future studies could also identify best practices that are applicable to a broader range of farming systems, contributing to sustainable improvements in animal welfare on a larger scale.

## 5. Conclusions

The findings of this study clearly demonstrate that targeted and continuous consultancy played a crucial role in improving animal welfare. Expert support not only allowed for the resolution of identified issues but also fostered a more strategic and informed management approach among farmers. The three key areas—farm management, infrastructure, and animal-based measures—are closely interconnected. Optimizing management practices and training farm personnel had a direct impact on animal welfare, without requiring immediate structural modifications. This indicates that, even in well-established farms, better resource utilization and more attentive management can lead to significant and tangible improvements.

The active involvement of farmers in the improvement process promoted a cultural shift, encouraging them to recognize animal welfare as a central element of their farm’s sustainability. This integrated approach enhances animal conditions while contributing to long-term economic and operational sustainability, making farms more efficient and competitive.

To ensure that these improvements are both effective and lasting, it is crucial that welfare assessments serve as a basis for ongoing, proactive management. Continuous and targeted support must accompany evaluation efforts, guiding farmers toward practical and sustainable solutions. The success of this system relies not only on identifying critical issues but, more importantly, on providing concrete tools to address and resolve them. Only through a structured and continuous consultancy framework can stable and long-term improvements can be achieved, thereby strengthening the link between animal welfare, production efficiency, and economic sustainability in the dairy sector.

Providing farmers with continuous training and tailored consultancy services based on their specific needs is essential. Stakeholders should support the development and funding of ongoing advisory programs and integrate these into broader animal welfare and farm sustainability initiatives. Moreover, fostering knowledge exchange networks between farmers and experts can amplify the positive effects observed.

Future research should evaluate the long-term sustainability of the improvements achieved, assessing whether positive outcomes persist beyond the consultancy period. It is also important to conduct cost–benefit analyses to quantify the economic impact of welfare improvements, supporting evidence-based policy decisions. Finally, studies on the scalability of these advisory models across different production systems and contexts will be crucial to promote wider adoption and improve animal welfare at both national and international levels. In this context, the integration of Precision Livestock Farming technologies, such as activity monitors and other sensor-based devices, offers promising opportunities for continuous, objective, and direct monitoring of animal welfare. These tools not only support farmers with real-time data for more precise and proactive interventions but also provide continuous assistance to consultants, making the advisory process more efficient and timely, and ultimately enhancing overall welfare and productivity outcomes.

## Figures and Tables

**Table 1:** Overall score of welfare assessments using the Classyfarm protocol for all farms (n.21).

Study Area	T0 ^a^	T1 ^a^	*p*-Value ^b^	Average Increase % ^c^
Total score	67.48 ± 4.75	82.05 ± 5.71	<0.0001	22
Area A. Management and personnel	61.33 ± 10.56	80.10 ± 6.35	<0.0001	34
Area B. Structures and facilities	63.05 ± 10.48	77.95 ± 8.22	<0.0001	26
Area C. Animal based measures ^d^	73.14 ± 5.98	85.29 ± 8.68	<0.0001	17
Area D. Major hazards	67.48 ± 8.23	69.71 ± 10.46	0.4173	4

^a^ Values are presented as mean ± standard deviation. ^b^ *p*-value was considered significant when *p* < 0.05. ^c^ The average increase was calculated using the formula = (T1 − T0/T0) × 100. ^d^ The number of animals observed for animal-based measures (ABMs) was determined based on herd size, as established by the Classyfarm Protocol, to ensure a statistically significant sample and accurate animal welfare assessment (Supplementary Table S3).

**Table 2:** Percentage of insufficient farms (scores below 60) observed in the first and second screenings recorded for the total and for each protocol area.

Study Area	Number of Insufficient Farms at T0	Number of Insufficient Farms at T1
Total score	0%	0%
Area A. Management and personnel	43%	0%
Area B. Structures and facilities	33%	0%
Area C. Animal based measures	5%	0%
Area D. Major hazards	14%	0%

**Table 3:** The main insufficiencies for animals older than 6 months at the time 0 expressed in the percentage of farms that were not sufficient for this item in relation to the total number of farms assessed (criteria considered insufficient for more than 20% of the total number of farms assessed, criteria that have not been observed in terms of legislative compliance, or criteria that have a greater impact on animal welfare).

Most Common Criticalities for Animals Older Than 6 Months					
Area	Item	Percentage of Insufficient Farms	Lactating cows	Dry cows	Heifers
Area A: Management and personnel	Hygiene, cleaning and management of housing and litter	24%	0%	40%	80%
	Cleaning of floors and other non-decubitus walkways	24%	40%	60%	40%
	Prevention of podal diseases	62%	No division by physiological phase ^b^		
	Water trough cleaning	38%	100%	75%	38%
Area B: Structures and equipment	Available surface for decubitus	62%	69%	100%	31%
	Size and operation of drinking troughs	38%	75%	75%	25%
	Infirmary	43%	No division by physiological phase ^b^		
Area C: Animal-based measures	Clean animals	100%	95%	76%	NE ^a^

^a^ NE: not evaluated. For that group, at that stage there is no assessment for that type of item. ^b^ Items for which division by physiological stage is not required.

**Table 4:** The main insufficiencies for animals younger than 6 months at time 0 expressed as percentage of farms that were not sufficient for this item in relation to the total number of farms assessed.

Increased Criticality for Animals Younger Than 6 Months				
Area	Item	Number of Insufficient Farms	Stage	
			Under 8 weeks of age	Over 8 weeks of age
Area A: Management and personnel	Hygiene, cleaning and management of housing and litter	5%		
Area B: Structures and equipment	Space availability for calves	9.5%	0%	100%
	Bedding material for newborn (less than 2 weeks of age)	9.5%	100%	NE ^a^
	Possibility for calves to see and touch each other (in single pens)	9.5% ^b^		

^a^ NE: not evaluated. For that group, at that stage there is no assessment for that type of item. ^b^ Criterion assessed only for animals in individual pens.

## Data Availability

The data presented in this study are available on request from the corresponding author.

## Supplementary Materials

The following supporting information can be downloaded at https://www.mdpi.com/article/10.3390/ani15152197/s1: Table S1: Average characteristics of the farms under study (n = 21): total number of animals by category, average milk yield, type of feed and housing system; Table S2: Classification of interventions according to their significance and effect on animal welfare, developed by the consulting experts; Figure S1. Distribution of total and partial welfare scores across 21 dairy farms at time points T0 and T1; Table S3: Minimum number of animals to be observed for the assessment of direct animal-based measures (ABMs), according to the Classyfarm protocol.

## References

[1] Croyle S.L., Belage E., Khosa D.K., LeBlanc S.J., Haley D.B., Kelton D.F. (2019). Dairy farmers’ expectations and receptivity regarding animal welfare advice: A focus group study. J. Dairy Sci..

[2] Kauppinen T., Valros A., Vesala K.M. (2013). Attitudes of dairy farmers toward cow welfare in relation to housing, management and productivity. Anthrozoos.

[3] Hansson H., Lagerkvist C.J. (2016). Dairy farmers’ use and non-use values in animal welfare: Determining the empirical content and structure with anchored best-worst scaling. J. Dairy Sci..

[4] Nash C.G.R., Kelton D.F., Vasseur E., Devries T.J., Parent D., Pellerin D., Carrier K., Pajor E.A., Rushen J., de Passillé A.M. (2019). A survey of practices implemented to improve cow comfort following an initial assessment on canadian dairy farms. Can. J. Anim. Sci..

[5] Booth N.J., Lloyd K. (1999). Stress in farmers. Int. J. Soc. Psychiatry.

[6] Firth H.M., Williams S.M., Herbison G.P., McGee R.O. (2007). Stress in New Zealand farmers. Stress Health.

[7] Ang H.B. (2010). Occupational Stress Among the New Zealand Farmers—A Review. Labour Employ. Work. N. Z..

[8] Balzani A., Hanlon A. (2020). Factors that Influence Farmers’ Views on Farm Animal Welfare: A Semi-Systematic Review and Thematic Analysis. Animals.

[9] Kallioniemi M.K., Kymäläinen H.R., Niemi J.K. (2024). Enabling factors and constraints for the adoption of animal welfare-enhancing technologies among Finnish dairy farmers. Front. Anim. Sci..

[10] Price J.L. (1997). Introduction Handbook of organizational measurement. Int. J. Manpow..

[11] Van der Doef M., Maes S. (1998). The job demand-control(-support) model and physical health outcomes: A review of the strain and buffer hypotheses. Psychol. Health.

[12] Häusser J.A., Mojzisch A., Niesel M., Shulz-Hardt S. (2010). Ten years on: A review of recent research on the Job Demand–Control (-Support) model and psychological well-being. Work Stress Int. J. Work Health Organ..

[13] Kaufmann G., Kaufmann A. (2009). Psychology in Organization and Leadership.

[14] Roter D.L., Stewart M., Putnam S.M., Stiles W., Inui T.S. (1997). Communication patterns of primary care physicians. JAMA.

[15] Cornell K.K., Kopcha M. (2007). Client-Veterinarian Communication: Skills for Client Centered Dialogue and Shared Decision Making. Vet. Clin. N. Am. Small Anim. Pract..

[16] Westermarck N. (1961). The human factor and economic progress. Indian J. Agric. Econ..

[17] Holm W., Lastein D.B. (2021). A Q study: Exploring the purpose of transdisciplinary dairy advisory services in Denmark. Acta Vet. Scand..

[18] Ritter C., Adams C.L., Kelton D.F., Barkema H.W. (2019). Factors associated with dairy farmers’ satisfaction and preparedness to adopt recommendations after veterinary herd health visits. J. Dairy Sci..

[19] (2023). Classifarm. www.Classyfarm.it.

[20] Checklist Classyfarm (2019). Checklist Classyfarm. Classyfarm Checklist Dairy Cattle Free Stall. Istituto Zooprofilattico Sperimentale della Lombardia e dell’Emilia Romagna; Universitá di Parma; Ministero della Salute. https://www.classyfarm.it/index.php/vet-aziendale-it.

[21] Mariottini F., Giuliotti L., Gracci M., Benvenuti M.N., Salari F., Arzilli L., Martini M., Roncoroni C., Brajon G. (2022). The ClassyFarm System in Tuscan Beef Cattle Farms and the Association between Animal Welfare Level and Productive Performance. Animals.

[22] Salari F., Roncoroni C., Mariottini F., Muzic A., Altomonte I., Sodi I., Creatini S., Giuliotti L., Brajon G., Martini M. (2024). Risk Categorization in On-Farm Welfare in Different-Sized Dairy Sheep Flocks. Animals.

[23] Bertocchi L., Fusi F., Angelucci A., Bolzoni L., Pongolini S., Strano R.M., Ginestreti J., Riuzzi G., Moroni P., Lorenzi V. (2018). Characterization of hazards, welfare promoters and animal-based measures for the welfare assessment of dairy cows: Elicitation of expert opinion. Prev. Vet. Med..

[24] President of Italian Repubblic D. Lgs. N. 146/2001. In *Legislative Decree n. 146 of 26 March 2001, Implementation of Directive 98/58/EC on the Protection of Animals on Farms [Decreto Legislativo n. 146 del 26 Marzo 2001, Attuazione della Direttiva 98/58/CE Relative alla Protezione degli Animali negli Allevamenti]*; Gazzetta Ufficiale 95 24/4/2001; 2001; Volume 180. https://www.normattiva.it/uri-res/N2Ls?urn:nir:stato:decreto.legislativo:2001-03-26;146.

[25] (2011). President of Italian Repubblic D. Lgs. n. 126/2011. In *Legislative Decree n. 126 of 7 July 2011, Implementation of Directive 2008/119/EC Which Establishes the Minimum Standards for the Protection of Calves [Decreto Legislativo n. 126 del 7 Luglio 2011, Attuazione della Direttiva 2008/119/CE che Stabilisce le Norme Minime per la Protezione dei Vitelli]*; Gazzetta Ufficiale 180 4/8/2011. https://www.normattiva.it/uri-res/N2Ls?urn:nir:stato:decreto.legislativo:2011-07-07;126!vig=.

[26] Tucker C.B., Weary D.M., von Keyserlingk M.A.G., Beauchemin K.A. (2009). Cow comfort in tie-stalls: Increased depth of shavings or straw bedding increases lying time. J. Dairy Sci..

[27] Tucker C.B., Weary D.M. (2004). Bedding on geotextile mattresses: How much is needed to improve cow comfort?. J. Dairy Sci..

[28] Reich L.J., Weary D.M., Veira D.M., Von Keyserlingk M.A.G. (2010). Effects of sawdust bedding dry matter on lying behavior of dairy cows: A dose-dependent response. J. Dairy Sci..

[29] Fulwider W.K., Grandin T., Garrick D.J., Engle T.E., Lamm W.D., Dalsted N.L., Rollin B.E. (2007). Influence of free-stall base on tarsal joint lesions and hygiene in dairy cows. J. Dairy Sci..

[30] Calamari L., Calegari F., Stefanini L. (2009). Effect of different free stall surfaces on behavioural, productive and metabolic parameters in dairy cows. Appl. Anim. Behav. Sci..

[31] Verbeke J., Piepers S., Supré K., De Vliegher S. (2014). Pathogen-specific incidence rate of clinical mastitis in Flemish dairy herds, severity, and association with herd hygiene. J. Dairy Sci..

[32] Doerfler R.L., Martin R., Bernhardt H. (2017). Implications of Robotic Walkway Cleaning for Hoof Disorders in Dairy Cattle. Int. J. Eng. Res. Appl..

[33] Gosling B. (2018). A review of cleaning and disinfection studies in farming environments. Livestock.

[34] Schütz K.E., Huddart F.J., Sutherland M.A., Stewart M., Cox N.R. (2015). Effects of space allowance on the behavior and physiology of cattle temporarily managed on rubber mats. J. Dairy Sci..

[35] Nielsen S.S., Alvarez J., Bicout D.J., Calistri P., Canali E., Drewe J.A., Garin-Bastuji B., Gonzales Rojas J.L., Gortázar Schmidt C., EFSA Panel on Animal Health and Animal Welfare (AHAW) (2023). Scientific Opinion: Welfare of dairy cows. EFSA J..

[36] Sutherland M.A., Worth G.M., Stewart M. (2014). The effect of rearing substrate and space allowance on the behavior and physiology of dairy calves. J. Dairy Sci..

[37] Relic R., Bojkovski J. (2010). Housing conditions in calves welfare risk assessment. J. Agric. Sci..

[38] Jensen M.B., Larsen L.E. (2014). Effects of level of social contact on dairy calf behavior and health. J. Dairy Sci..

[39] Van der Spek D. (2015). Genetic Background of Claw Health in Dairy Cattle. Ph.D. Thesis.

[40] Van der Linde C., de Jong G., Koenen E.P.C., Eding H. (2010). Claw health index for Dutch dairy cattle based on claw trimming and conformation data. J. Dairy Sci..

[41] Van Der Waaij E.H., Holzhauer M., Ellen E., Kamphuis C., De Jong G. (2005). Genetic parameters for claw disorders in Dutch Dairy Cattle and correlations with conformation traits. J. Dairy Sci..

[42] Alvergnas M., Strabel T., Rzewuska K., Sell-Kubiak E. (2019). Claw disorders in dairy cattle: Effects on production, welfare and farm economics with possible prevention methods. Livest. Sci..

[43] Leach K.A., Whay H.R., Maggs C.M., Barker Z.E., Paul E.S., Bell A.K., Main D.C.J. (2010). Working towards a reduction in cattle lameness: 1. Understanding barriers to lameness control on dairy farms. Res. Vet. Sci..

[44] Schütz K.E., Huddart F.J., Cox N.R. (2019). Manure contamination of drinking water influences dairy cattle water intake and preference. Appl. Anim. Behav. Sci..

[45] Willms W.D., Kenzie O.R., Mcallister T.A., Colwell D., Veira D., Wilmshurst J.F., Entz T., Olson M.E. (2002). Society for Range Management Effects of Water Quality on Cattle Performance. J. Range Manag..

[46] Lardner H.A., Kirychuk B.D., Braul L., Willms W.D., Yarotski J. (2005). The effect of water quality on cattle performance on pasture. Aust. J. Agric. Res..

[47] Wilks D.L., Coppock C.E., Lanham J.K., Brooks K.N., Baker C.C., Bryson W.L., Elmore R.G., Stermer R.A. (1990). Responses of Lactating Holstein Cows to Chilled Drinking Water in High Ambient Temperatures. J. Dairy Sci..

[48] Utley P.R., Bradley N.W., Boling J.A. (1970). Effect of restricted water intake on feed intake, nutrient digestibility and nitrogen metabolism in steers. J. Anim. Sci..

[49] Little W., Sansom B.F., Manston R., Allen W.M. (1978). The effects of reducing the water intake of lactating dairy cows by 40% for 3 weeks. Anim. Sci..

[50] Little W., Collis K.A., Gleed P.T., Sansom B.F., Allen W.M., Quick A.J. (1980). Effect of reduced water intake by lactating dairy cows on behaviour, milk yield and blood composition. Vet. Rec..

[51] Farm Animal Welfare Council (1992). FAWC updates the five freedoms. Vet. Rec..

[52] European Commission (1998). Directive 98/58/EC. Council Directive concerning the protection of animals kept for farming purposes. Off. J. Eur. Union.

