# Supplementary material for: Effectiveness of Targeted Advisory Interventions in Enhancing Welfare on Dairy Farms
Source: Animals (Basel). 2025 Jul 25;15(15):2197. doi: 10.3390/ani15152197 (PMC12345549; doi:10.3390/ani15152197)
Supplement: Supplementary file 1 [file animals-15-02197-s001.zip › animals-3750023-supplementary.pdf]

SUPPLEMENTARY TABLES

Table S1. Average characteristics of the farms under study (n = 21): total number of animals by category, average milk yield, type of feed, and housing system

Farm Characteristic	Parameter	T0	T1
Total number of animals	Mean ± SD	159.52 ± 165.87	155.48 ± 164.44
	Range	(27-652)	(26-630)
Number of lactating Cow	Mean ± SD	74.76 ± 76.26	73.76 ± 75.16
	Range	(12-303)	(12-285)
Number of dry cow	Mean ± SD	15.00 ± 15.60	14.52 ± 15.55
	Range	(0-57)	(2-63)
Young stock > 6 month	Mean ± SD	46,00 ± 56,60	48,62 ± 57,30
	Range	(8-219)	(5-214)
Calves	Mean ± SD	23.76 ± 22.83	18.57 ± 19.68
	Range	(3-88)	(0-68)
Milk yeald (kg milk/head/day)	Mean ± SD	25.67 ± 5.19	26.48 ± 5.17
	Range	(14-35)	(17-36)
Type of housing		Free housing	Free housing

Table S2. Classification of interventions according to their significance and effect on animal welfare, developed by the consulting experts

Intervention	Importance
Mandatory interventions for self-monitoring and official control	Resolution of possible legislative non-conformities
Minimal improvement	Small managerial or structural interventions, aimed at improving conditions and well-being scores
Future interventions, which can be performed later	Interventions to be considered for possible future improvements/investments

Figure S1. Distribution of total and partial welfare scores across 21 dairy farms at time points T0 and T1. Boxplots represent the median, interquartile range, and range of scores for total welfare and individual areas (management and personnel, structures and facilities, animal-based measures, major hazards) assessed according to the protocol. This figure illustrates the variability and improvement trends between the initial and follow-up evaluations.

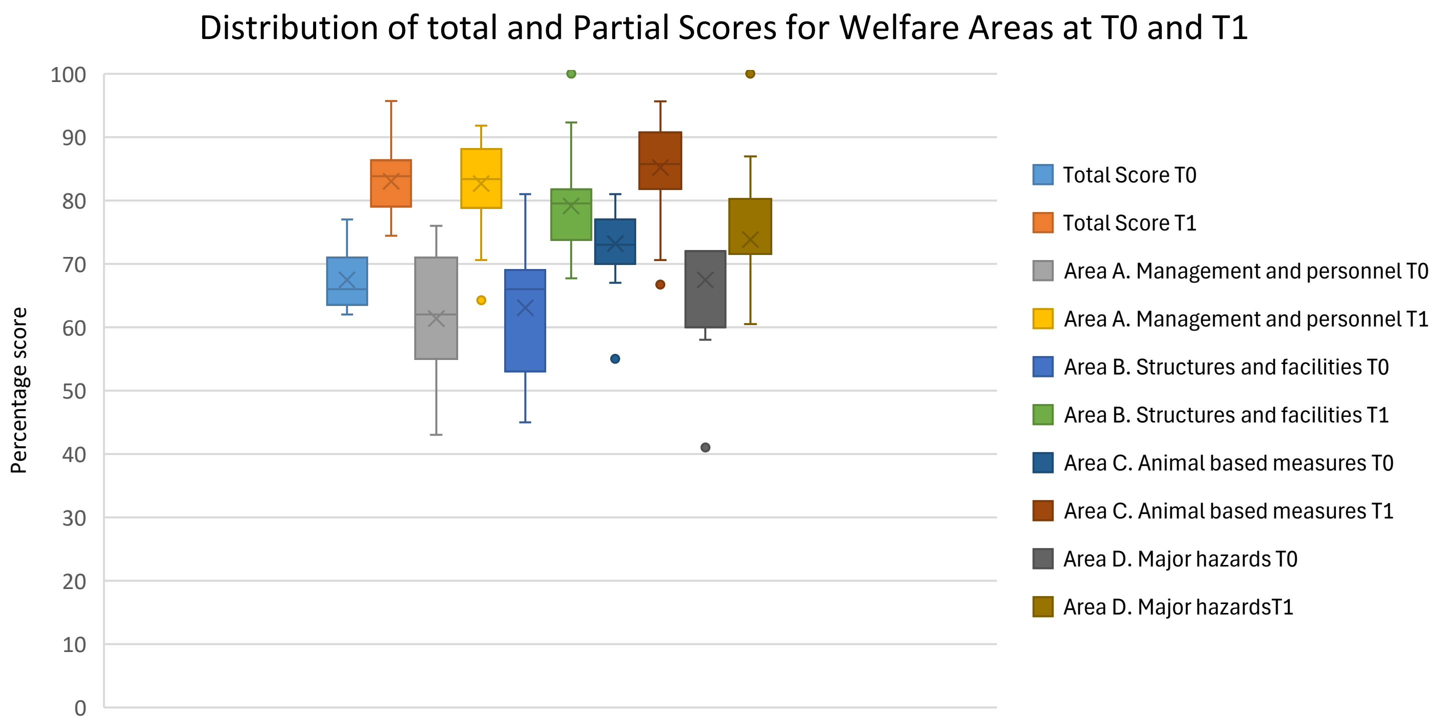

Table S3. Minimum number of animals to be observed for the assessment of direct animal-based measures (ABMs), according to the Classyfarm protocol.

To ensure accurate operation and assess the condition of the herd, a minimum number of statistically significant animals must be observed in the case of direct ABMs, and this number should be proportional to the size of the group. (Classyfarm Protocol of Dairy Cow)

**Size of the group**	**Minimum number of animals to be observed**
≤ 30	All animals
≥ 31 ≤99	30-39
≥ 100 ≤ 199	40-50
≥ 200 ≤ 299	51-55
≥ 300 ≤ 549	55-59
≥ 550 ≤1000	60-63
≥ 1001 ≤3000	63-65
